# Functions and Disorders of the Reproductive Organs in Childhood, Youth, Adult Age and Advanced Life

**Published:** 1872-01

**Authors:** 


*Functions and Disorders of the Reproductive Organs, in Childhood, Youth, Adult Age and Advanced Life.* By William Acton, M. R. C. S. Philadelphia, Lindsay & Blackiston, 1871.

This work is, as it should be, largely in the hands of the profession; it is a complete and scientific treatise upon the disorders and functions of the reproductive organs, and as such should be in the hands of all physicians. There is not a single class of diseases so imperfectly understood and so unscientifically treated as those of the reproductive organs. Quacks and charlatans have educated the people in error to the highest degree possible, their fears and apprehensions have been cultivated and finally they have been imposed upon and cheated. This book has done much to correct the views of the profession and to improve their modes of treating many of the diseases and derangements of the reproductive organs. It will be well for the profession and public if this work is carefully read and considered; it is suggestive of much truth with which all physicians should be acquainted.

